# Correction: Burden of disease and risk factors for mortality amongst hospitalized newborns in Nigeria and Kenya

**DOI:** 10.1371/journal.pone.0306684

**Published:** 2024-07-01

**Authors:** Helen M. Nabwera, Dingmei Wang, Olukemi O. Tongo, Pauline E. A. Andang’o, Isa Abdulkadir, Chinyere V. Ezeaka, Beatrice N. Ezenwa, Iretiola B. Fajolu, Zainab O. Imam, Martha K. Mwangome, Dominic D. Umoru, Abimbola E. Akindolire, Walter Otieno, Grace M. Nalwa, Alison W. Talbert, Ismaela Abubakar, Nicholas D. Embleton, Stephen J. Allen, on behalf of the Neonatal Nutrition Network (NeoNuNet)

The first and second authors, Helen M. Nabwera and Dingmei Wang should be noted as joint first co-authors of this work.
