# Supplementary material for: Burden of disease and risk factors for mortality amongst hospitalized newborns in Nigeria and Kenya
Source: PLoS One. 2021 Jan 14;16(1):e0244109. doi: 10.1371/journal.pone.0244109 (PMC7808658; doi:10.1371/journal.pone.0244109)
Supplement: S1 File — (DOCX) [file pone.0244109.s002.docx]

| **Supplementary Tables**  **S1 Table: Maternal and newborn variables according to country and level of neonatal care** | | | | | | | | | | |
| --- | --- | --- | --- | --- | --- | --- | --- | --- | --- | --- |
| **Description** | **Total** |  | **Country** | | |  | **Level of neonatal care** | | |  |
|  | **N= 2280** |  | **NIGERIA N=1404** | **KENYA**  **N=876** | ***P*** |  | ***Tertiary***  **N=1888** | ***Secondary***  ***N=392*** | ***P*** |  |
| **Maternal details** |  |  |  |  |  |  |  |  |  |  |
| **Maternal age in years, n**  **mean (SD)** | 2215  28.7 (6.2) |  | 1402  30.5 (5.8) | 813  25.6 (5.7) | <0.001 |  | 1826  29.1 (6.2) | 389  27.0 (6.2) | <0.001 |  |
| <18, n (%) | 48 (2.2) |  | 7 (0.5) | 41 (5.04) |  |  | 30 (1.6) | 18 (4.6) |  |  |
| 18-29, n (%) | 1164 (52.5) |  | 593 (42.3) | 571 (70.2) |  |  | 924 (50.6) | 240 (61.7) |  |  |
| >=30, n (%) | 1003 (45.3) |  | 802 (57.2) | 201 (24.7) |  |  | 872 (47.8) | 131 (33.7) |  |  |
|  |  |  |  |  |  |  |  |  |  |  |
| **Maternal education, n** | 2146 |  | 1381 | 765 | <0.001 |  | 1759 | 387 | <0.001 |  |
| Did not complete primary education, n (%) | 297 (13.9) |  | 88 (6.4) | 209 (27.3) |  |  | 169 (9.6) | 128 (33.1) |  |  |
| Completed only primary school, n (%) | 621 (28.9) |  | 360 (26.1) | 261 (34.1) |  |  | 517 (29.4) | 104 (26.9) |  |  |
| Completed secondary school, n (%) | 533 (24.8) |  | 363 (26.3) | 170 (22.2) |  |  | 488 (27.7) | 45 (11.6) |  |  |
| Completed tertiary level education, n (%) | 695 (36.4) |  | 570 (41.2) | 125 (16.4) |  |  | 585 (33.3) | 110 (28.4) |  |  |
|  |  |  |  |  |  |  |  |  |  |  |
| **Maternal occupation, n** | 2155 |  | 1383 | 772 | <0.001 |  | 1767 | 388 | <0.001 |  |
| Unemployed or housewife, n (%) | 809 (37.5) |  | 326 (23.6) | 483 (62.6) |  |  | 587 (33.2) | 222 (57.2) |  |  |
| Petty trader/ labourer, n (%) | 680 (31.5) |  | 496 (35.9) | 184 (23.8) |  |  | 598 (33.8) | 82 (21.1) |  |  |
| Junior schools teachers/drivers, n (%) | 320 (14.9) |  | 264 (19.1) | 56 (7.3) |  |  | 290 (16.4) | 30 (7.7) |  |  |
| Intermediate public servant/senior school teachers, n (%) | 187 (8.7) |  | 157 (11.4) | 30 (3.9) |  |  | 161 (9.1) | 26 (6.7) |  |  |
| Senior public servant/ professionals/ large scale traders, n (%) | 159 (7.4) |  | 140 (10.0) | 19 (2.4) |  |  | 131 (7.4) | 28 (7.2) |  |  |
|  |  |  |  |  |  |  |  |  |  |  |
| **Marital status, n** | 2188 |  | 1378 | 810 | <0.001 |  | 1809 | 379 | 0.748 |  |
| Single, n (%) | 175 (8.0) |  | 33 (2.4) | 142 (17.6) |  |  | 143 (7.9) | 32 (8.4) |  |  |
| Married, n (%) | 2004 (91.6) |  | 1342 (97.4) | 662 (81.7) |  |  | 1659 (91.7) | 345 (91.0) |  |  |
| Divorced, n (%) | 9 (0.4) |  | 3 (0.2) | 6 (0.7) |  |  | 7 (0.4) | 2 (0.6) |  |  |
|  |  |  |  |  |  |  |  |  |  |  |
| **Parity, n** | 2216 |  | 1401 | 815 | <0.001 |  | 1824 | 392 | <0.001 |  |
| 1, n (%) | 97 (4.4) |  | 94 (6.7) | 3 (0.4) |  |  | 97 (5.3) | 0 |  |  |
| 2, n (%) | 624 (28.2) |  | 350 (25.0) | 274 (33.6) |  |  | 506 (27.7) | 118 (30.1) |  |  |
| >2, n (%) | 1495 (67.5) |  | 957 (68.3) | 538 (66.0) |  |  | 1221 (67.0) | 274 (69.9) |  |  |
|  |  |  |  |  |  |  |  |  |  |  |
| **Number of stillbirths, n** | 2222 |  | 1401 | 821 | 0.553 |  | 1830 | 392 | 0.061 |  |
| None, n (%) | 2066 (93.0) |  | 1305 (93.2) | 761 (92.7) |  |  | 1712 (93.6) | 354 (90.3) |  |  |
| One, n (%) | 118 (5.3) |  | 70 (5.0) | 48 (5.9) |  |  | 88 (4.8) | 30 (7.7) |  |  |
| Two or more, n (%) | 38 (1.7) |  | 26 (1.8) | 12 (1.4) |  |  | 30 (1.6) | 8 (2.0) |  |  |
|  |  |  |  |  |  |  |  |  |  |  |
| **Antenatal clinic visits, n** | 2140 |  | 1383 | 757 | <0.001 |  | 1752 | 388 | 0.002 |  |
| Zero to three, n (%) | 824 (38.5) |  | 502 (36.3) | 322 (42.5) |  |  | 670 (38.2) | 154 (39.7) |  |  |
| Four to seven, n (%) | 1073 (50.1) |  | 657 (47.5) | 416 (55.0) |  |  | 863 (49.3) | 210 (54.1) |  |  |
| Eight or more, n (%) | 243 (11.4) |  | 224 (16.2) | 19 (2.5) |  |  | 219 (12.5) | 24 (6.2) |  |  |
|  |  |  |  |  |  |  |  |  |  |  |
| **Number of foetuses, n** | 2267 |  | 1398 | 869 | <0.001 |  | 1875 | 392 | 0.004 |  |
| 1, n (%) | 1825 (80.5) |  | 1111 (79.5) | 714 (82.2) |  |  | 1494 (79.7) | 331 (84.5) |  |  |
| 2, n (%) | 337 (14.9) |  | 200 (14.3) | 137 (15.8) |  |  | 282 (15.0) | 55 (14.0) |  |  |
| 3- 5, n (%) | 105 (4.6) |  | 87 (6.2) | 18 (2.0) |  |  | 99 (5.3) | 6 (1.5) |  |  |
|  |  |  |  |  |  |  |  |  |  |  |
| **HIV status, n** | 2039 |  | 1204 | 835 | <0.001 |  | 1674 | 365 | 0.018 |  |
| Positive, n (%) | 128 (6.3) |  | 25 (2.1) | 103 (12.3) |  |  | 115 (6.9) | 13 (3.4) |  |  |
|  |  |  |  |  |  |  |  |  |  |  |
| **Hepatitis B, n** | 1079 |  | 1061 | 18 | 0.999 |  | 987 | 92 | 0.999 |  |
| Positive, n (%) | 25 (2.3) |  | 25 (2.4) | 0 |  |  | 23(2.3) | 2 (2.2) |  |  |
|  |  |  |  |  |  |  |  |  |  |  |
| **Syphilis, n** | 1388 |  | 632 | 756 | 0.504 |  | 1043 | 345 | 0.999 |  |
| Positive, n (%) | 2 (0.1) |  | 0 | 2 (0.3) |  |  | 2 (0.2) | 0 |  |  |
|  |  |  |  |  |  |  |  |  |  |  |
| **Gestational diabetes, n** | 1755 |  | 979 | 776 | <0.001 |  | 1419 | 336 | 0.047 |  |
| Yes, n (%) | 34 (1.9) |  | 31 (3.2) | 3 (0.4) |  |  | 32 (2.3) | 2 (0.6) |  |  |
|  |  |  |  |  |  |  |  |  |  |  |
| **Pregnancy induced hypertension, n** | 2136 |  | 1334 | 802 | <0.001 |  | 1778 | 358 | <0.001 |  |
| Yes, n (%) | 363 (17.0) |  | 293 (22.0) | 70 (8.7) |  |  | 332 (18.7) | 31 (8.7) |  |  |
|  |  |  |  |  |  |  |  |  |  |  |
| **Antepartum haemorrhage, n** | 2184 |  | 1374 | 810 | 0.005 |  | 1819 | 365 | 0.842 |  |
| Yes, n (%) | 161 (7.4) |  | 118 (8.6) | 43 (6.3) |  |  | 135(7.4) | 26 (7.1) |  |  |
|  |  |  |  |  |  |  |  |  |  |  |
| **Details of labour and delivery** |  |  |  |  |  |  |  |  |  |  |
| **Place of delivery, n** | 2280 |  | 1404 | 876 | 0.009 |  | 1888 | 392 | 0.266 |  |
| Health facility, n (%) | 2128 (93.3) |  | 1297 (92.4) | 831 (94.9) |  |  | 1768 (93.6) | 360 (91.8) |  |  |
| Home, n (%) | 99 (4.4) |  | 64 (4.6) | 35 (4.0) |  |  | 76 (4.0) | 23 (5.9) |  |  |
| Other, n (%)* | 53 (2.3) |  | 43 (3.1) | 10 (1.1) |  |  | 4 4(2.3) | 9 (2.3) |  |  |
|  |  |  |  |  |  |  |  |  |  |  |
| **Prolonged rupture of membranes ≥ 18 hours, n** | 2151 |  | 1384 | 767 | <0.001 |  | 1769 | 382 | 0.002 |  |
| Yes, n (%) | 359 (16.7) |  | 280 (20.2) | 79 (10.3) |  |  | 316 (17.9) | 43 (11.3) |  |  |
|  |  |  |  |  |  |  |  |  |  |  |
| **Maternal peripartum fever, n** | 2159 |  | 1366 | 793 | 0.323 |  | 1778 | 381 | 0.005 |  |
| Confirmed or suspected, n (%) | 204 (9.5) |  | 132 (9.7) | 72 (8.1) |  |  | 158 (8.9) | 45 (12.1) |  |  |
|  |  |  |  |  |  |  |  |  |  |  |
| **Mother treated with antibiotics within 24 hrs before/after birth, n** | 2147 |  | 1298 | 849 | <0.001 |  | 1776 | 371 | <0.001 |  |
| Yes, n (%) | 1263 (58.8) |  | 688 (53.0) | 575 (67.7) |  |  | 1153 (64.9) | 110 (29.7) |  |  |
|  |  |  |  |  |  |  |  |  |  |  |
| **Mother <37 gestational weeks received antenatal dexamethasone, n** | 1083 |  | 759 | 324 | <0.001 |  | 945 | 138 | <0.001 |  |
| 4 doses, n (%) | 115 (10.6) |  | 99 (13.0) | 16 (4.9) |  |  | 112 (11.9) | 3 (2.2) |  |  |
| 1-3 doses, n (%) | 171 (15.8) |  | 161 (21.2) | 10 (3.1) |  |  | 163 (17.2) | 8 (5.8) |  |  |
| None, n (%) | 797 (73.6) |  | 499 (65.8) | 298 (92.0) |  |  | 670 (70.9) | 127 (92.0) |  |  |
|  |  |  |  |  |  |  |  |  |  |  |
| **Mode of delivery, n** | 2277 |  | 1402 | 875 | <0.001 |  | 1886 | 391 | <0.001 |  |
| CS, n (%) | 1006 (44.2) |  | 723 (51.6) | 283 (32.3) |  |  | 879 (46.6) | 127 (32.5) |  |  |
| Vaginal assisted, n (%) | 83 (3.6) |  | 45 (3.2) | 38 (4.3) |  |  | 57 (3.0) | 26 (6.7) |  |  |
| Vaginal unassisted, n (%) | 1188 (52.2) |  | 634 (45.2) | 554 (63.3) |  |  | 950 (50.4) | 238 (60.8) |  |  |
|  |  |  |  |  |  |  |  |  |  |  |
| **Maternal outcome, n** | 2267 |  | 1398 | 0 | <0.001 |  | 1875 | 392 | 0.104 |  |
| Maternal death, n (%) | 24 (1.1) |  | 24 (1.7) | 0 |  |  | 23 (1.2) | 1 (0.3) |  |  |
|  |  |  |  |  |  |  |  |  |  |  |
| **Newborn details** |  |  |  |  |  |  |  |  |  |  |
| **Gender, n** | 2280 |  | 1404 | 876 | 0.061 |  | 1888 | 392 | 0.019 |  |
| Male, n (%) | 1292 (56.7) |  | 774 (55.1) | 518 (59.1) |  |  | 1049 (55.6) | 243 (62.0) |  |  |
|  |  |  |  |  |  |  |  |  |  |  |
| **Birth weight, kg, mean (SD)** | 2182  2.3 (0.9) |  | 1322  2.2 (0.9) | 860  2.5 (0.9) | <0.001 |  | 1807  2.3 (0.9) | 375  2.5 (0.9) | <0.001 |  |
| <1, n (%) | 107 (4.9) |  | 70 (5.3) | 37 (4.3) |  |  | 92 (5.1) | 15 (4.0) |  |  |
| 1-<1.5, n (%) | 373 (17.1) |  | 252 (19.0) | 121 (14.1) |  |  | 319 (17.7) | 54 (14.4) |  |  |
| 1.5-<2.5, n (%) | 734 (33.6) |  | 497 (37.6) | 237 (27.6) |  |  | 642 (35.5) | 92 (24.5) |  |  |
| 2.5-<4, n (%) | 909 (41.7) |  | 466 (35.3) | 443 (51.5) |  |  | 705 (39.0) | 204 (54.4) |  |  |
| 4-5.5, n (%) | 59 (2.7) |  | 37 (2.8) | 22 (2.7) |  |  | 49 (2.7) | 10 (2.7) |  |  |
|  |  |  |  |  |  |  |  |  |  |  |
| **Gestation, weeks, median (IQR)** | 2236  36 (32, 39) |  | 1394  35 (32, 38) | 842  37.6 (33, 39.1) | <0.001 |  | 1846  36 (32, 38) | 390  38 (33.6, 40) | <0.001 |  |
| <28, n (%) | 119 (5.3) |  | 77 (5.5) | 42 (5.0) |  |  | 105 (5.7) | 14 (3.6) |  |  |
| 28-<32, n (%) | 364 (16.3) |  | 267 (19.2) | 97 (11.5) |  |  | 319 (17.3) | 45 (11.5) |  |  |
| 32-<37, n (%) | 689 (30.8) |  | 456 (32.7) | 233 (27.7) |  |  | 597 (32.3) | 92 (23.6) |  |  |
| 37-42, n (%) | 981 (43.9) |  | 573 (41.1) | 408 (48.5) |  |  | 780 (42.3) | 201 (51.6) |  |  |
| 42-45, n (%) | 83 (3.7) |  | 21 (1.5) | 62 (7.4) |  |  | 45 (2.4) | 38 (9.7) |  |  |
|  |  |  |  |  |  |  |  |  |  |  |
| **Method used to assess gestation, n** | 2245 |  | 1397 | 848 | <0.001 |  | 1854 | 391 | <0.001 |  |
| Ballard or other charts, n (%) | 425 (18.9) |  | 172 (12.3) | 253 (29.8) |  |  | 186 (10.0) | 239 (61.1) |  |  |
| Early USS, n (%) | 233 (10.4) |  | 226 (16.2) | 7 (0.8) |  |  | 227 (12.3) | 6 (1.5) |  |  |
| Maternal last menstrual period, n (%) | 1587 (70.7) |  | 999 (71.5) | 588 (69.4) |  |  | 1441 (77.7) | 146 (37.4) |  |  |
|  |  |  |  |  |  |  |  |  |  |  |
| **Admitted to NNU from, n** | 2280 |  | 1404 | 876 | <0.001 |  | 1888 | 392 | <0.001 |  |
| Home, n (%) | 131 (5.8) |  | 104 (7.4) | 27 (3.1) |  |  | 99 (5.2) | 32 (8.2) |  |  |
| Labour ward, n (%) | 910 (39.9) |  | 606 (43.2) | 304 (34.7) |  |  | 800 (42.4) | 110 (28.1) |  |  |
| Postnatal ward, n (%) | 202 (8.9) |  | 76 (5.4) | 126 (14.4) |  |  | 102 (5.4) | 100 (25.5) |  |  |
| Health facility, n (%) | 660 (28.9) |  | 391 (27.9) | 269 (30.7) |  |  | 531 (28.1) | 129 (32.9) |  |  |
| Other, n (%)^**^ | 377 (16.5) |  | 227 (16.1) | 150 (17.1) |  |  | 356 (18.9) | 21 (5.3) |  |  |
|  |  |  |  |  |  |  |  |  |  |  |
| **Prophylactic antibiotics, n** | 2252 |  | 1393 | 859 | <0.001 |  | 1864 | 388 | <0.001 |  |
| Yes, n (%) | 1113 (49.4) |  | 963 (69.1) | 150 (17.5) |  |  | 1014 (54.4) | 99 (25.5) |  |  |
|  |  |  |  |  |  |  |  |  |  |  |
| **Congenital anomalies, n** | 2269 |  | 1395 | 874 | 0.070 |  | 1877 | 392 | 0.611 |  |
| Yes, n (%) | 128 (5.6) |  | 69 (5.0) | 59 (6.8) |  |  | 108 (5.8) | 20 (5.1) |  |  |
|  |  |  |  |  |  |  |  |  |  |  |
| **Congenital heart diseases, n** | 2234 |  | 1368 | 866 | 0.001 |  | 1843 | 391 | 0.048 |  |
| Yes, n (%) | 56 (2.5) |  | 46 (3.4) | 10 (1.2) |  |  | 52 (2.8) | 4 (1.0) |  |  |
|  |  |  |  |  |  |  |  |  |  |  |
| **Patent ductus arteriosus among infant birth weight<1.5kg, n** | 468 |  | 318 | 150 | 0.068 |  | 400 | 68 | 0.488 |  |
| Yes, n (%) | 17 (3.6) |  | 15 (4.7) | 2 (1.3) |  |  | 16 (4.0) | 7 (1.5) |  |  |
|  |  |  |  |  |  |  |  |  |  |  |
| **Received phototherapy, n** | 2262 |  | 1394 | 868 | <0.001 |  | 1871 | 391 | <0.001 |  |
| Yes, n (%) | 987 (43.6) |  | 881 (63.2) | 106 (12.2) |  |  | 870 (46.5) | 117 (29.9) |  |  |
|  |  |  |  |  |  |  |  |  |  |  |
| **Other common morbidities^***^** | 2280 |  | 1404 | 876 |  |  | 1888 | 392 |  |  |
| Asphyxia, n (%) | 547 (24.0) |  | 314 (22.4) | 233 (26.6) | 0.021 |  | 477 (25.3) | 70 (17.9) | 0.002 |  |
| Respiratory conditions, n (%) | 817 (35.8) |  | 416 (29.6) | 401 (45.8) | <0.001 |  | 716 (37.9) | 101 (25.8) | <0.001 |  |
| Abdominal condition, n (%) | 71 (3.1) |  | 63 (4.5) | 8 (0.9) | <0.001 |  | 63 (3.3) | 8 (2.0) | 0.179 |  |
| Suspected sepsis, n (%) | 955 (41.9) |  | 443 (31.6) | 512 (58.5) | <0.001 |  | 769 (40.7) | 186 (47.5) | 0.014 |  |
|  |  |  |  |  |  |  |  |  |  |  |
| **Infant final outcome, n** | 2262 |  | 1398 | 864 | 0.019 |  | 1870 | 392 | <0.001 |  |
| Absconded/discharge against medical, n (%) | 42 (1.9) |  | 33 (2.4) | 9 (1.0) |  |  | 32 (1.7) | 10 (2.6) |  |  |
| Died, n (%) | 423 (18.7) |  | 265 (19.0) | 158 (18.3) |  |  | 376 (20.1) | 47 (12.0) |  |  |
| Discharged home with morbidities, n (%) | 55 (2.4) |  | 31 (2.2) | 24 (2.8) |  |  | 37 (2.0) | 18 (4.6) |  |  |
| Discharged home with no morbidities, n (%) | 1699 (75.1) |  | 1035 (74.0) | 664 (76.9) |  |  | 1387 (74.2) | 312 (79.6) |  |  |
| Transferred out, n (%) | 43 (1.9) |  | 34 (2.4) | 9 (1.0) |  |  | 38 (2.0) | 5 (1.2) |  |  |
|  |  |  |  |  |  |  |  |  |  |  |
| **Timing of mortality in infants, n** | 421 |  | 263 | 158 | 0.095 |  | 374 | 47 | 0.478 |  |
| Age at time of death in days, median (IQR) | 2 (1, 5) |  | 3 (1, 5) | 1 (1, 3) |  |  | 2 (1, 5) | 2 (1, 5) |  |  |
| < 7 days, n (%) | 335 (79.6) |  | 202 (76.8) | 133 (84.2) |  |  | 299 (80.0) | 36 (76.6) |  |  |
| 7-<14 days, n (%) | 59 (14.0) |  | 45 (17.1) | 14 (8.9) |  |  | 54 (14.4) | 5 (10.6) |  |  |
| 15-30 days, n (%) | 23 (5.5) |  | 12 (4.6) | 11 (6.9) |  |  | 17 (4.6) | 3 (12.8) |  |  |
| >30 days, n (%) | 4 (0.9) |  | 4 (1.5) | 0 |  |  | 4 (1.0) | 0 |  |  |
|  |  |  |  |  |  |  |  |  |  |  |
| **Final outcome among infants <1.5kg, n** | 472 |  | 318 | 154 | 0.913 |  | 403 | 69 | 0.024 |  |
| Absconded/discharge against medical, n (%) | 3 (0.6) |  | 2 (0.6) | 1 (0.6) |  |  | 2 (0.5) | 1 (1.5) |  |  |
| Died, n (%) | 222 (47.0) |  | 151 (47.5) | 71 (46.1) |  |  | 200 (49.6) | 22 (21.8) |  |  |
| Discharged home with morbidities, n (%) | 4 (0.9) |  | 3 (0.9) | 1 (0.7) |  |  | 3 (0.7) | 1 (1.5) |  |  |
| Discharged home with no morbidities, n (%) | 237 (50.2) |  | 157 (49.4) | 80 (51.9) |  |  | 192 (47.6) | 45 (65.2) |  |  |
| Transferred out, n (%) | 6 (1.3) |  | 5 (1.6) | 1 (0.7) |  |  | 6 (1.5) | 0 |  |  |
|  |  |  |  |  |  |  |  |  |  |  |
| **Timing of mortality in infants with birth weight <1.5kg, n** | 221 |  | 150 | 71 | 0.456 |  | 199 | 22 | 0.126 |  |
| Age at time of death in days, median (IQR) | 2 (1, 6) |  | 3 (1, 5) | 1 (1, 7) |  |  | 2 (1, 5) | 3 (1, 16) |  |  |
| < 7 days, n (%) | 165 (74.7) |  | 114 (76.0) | 51 (71.8) |  |  | 151 (75.9) | 14 (63.6) |  |  |
| 7-<14 days, n (%) | 33 (14.9) |  | 23 (15.3) | 10 (14.1) |  |  | 31 (15.6) | 2 (9.1) |  |  |
| 15-30 days, n (%) | 19 (8.6) |  | 9 (6.0) | 10 (14.1) |  |  | 13 (6.5) | 6 (27.3) |  |  |
| >30 days, n (%) | 4 (1.8) |  | 4 (2.7) | 0 |  |  | 4 (2.0) | 0 |  |  |
| Note: *Top three: 20 mission home, 20 traditional birth attendant home, 7 delivered on the way to hospital; ^**^1172 mothers are <37 gestational weeks; ^**^348 from theatre, and 22 from the clinics or wards | | | | | | | | | | |
|  | | | | | | | | | | |

| **S2 Table: Univariate logistic regression analysis of factors associated with neonatal mortality** | | | | |
| --- | --- | --- | --- | --- |
| **Risk factors** |  | ***Odds Ratio*** | ***P value*** | ***95% Confidence Interval*** |
| **Maternal factors** |  |  |  |  |
| **Maternal age (>=30)** |  | 1.04 | 0.716 | 0.83, 1.29 |
| **Maternal education** |  |  |  |  |
| Did not complete primary education |  | 1 |  |  |
| Completed only primary school |  | 1.27 | 0.166 | 0.90, 1.81 |
| Completed secondary school |  | 0.97 | 0.877 | 0.67, 1.40 |
| Completed tertiary level education |  | **0.64** | **0.017** | **0.44, 0.92** |
| **Maternal occupation** |  |  |  |  |
| Unemployed or housewife |  | 1 |  |  |
| Petty trader/ labourer |  | 1.11 | 0.419 | 0.86, 1.44 |
| Junior schoolteacher/driver |  | 0.93 | 0.683 | 0.66, 1.30 |
| Intermediate public servant/senior schoolteacher |  | **0.55** | **0.018** | **0.34, 0.90** |
| Senior public servant/ professionals/ large scale traders |  | **0.53** | **0.018** | **0.31, 0.90** |
| **Marital status** |  |  |  |  |
| Single |  | 1 |  |  |
| Divorced |  | 0.43 | 0.436 | 0.04, 3.57 |
| Married |  | 0.75 | 0.137 | 0.52, 1.1 |
| **Number of stillbirths** |  |  |  |  |
| None |  | 1 |  |  |
| One |  | 0.85 | 0.525 | 0.51, 1.40 |
| Two or more |  | 1.36 | 0.426 | 0.64, 3.00 |
| **Antenatal clinic visits** |  |  |  |  |
| Zero to three |  | 1 |  |  |
| Four to seven |  | **0.49** | **<0.001** | **0.38, 0.61** |
| Eight or more |  | **0.33** | **<0.001** | **0.21, 0.52** |
| **Number of foetuses** |  |  |  |  |
| 1 |  | 1 |  |  |
| 2 |  | **1.53** | **0.002** | **1.16, 2.02** |
| 3- 5 |  | 1.34 | 0.222 | 0.84, 2.18 |
| **HIV status (positive)** |  | 0.65 | 0.116 | 0.38, 1.11 |
| **Hepatitis B (positive)** |  | 0.88 | 0.821 | 0.30, 2.60 |
| **Syphilis (positive)** |  | 6.02 | 0.205 | 0.38, 96.6 |
| **Gestational diabetes (yes)** |  | 0.33 | 0.130 | 0.08, 1.38 |
| **Pregnancy induced hypertension (yes)** |  | 1.02 | 0.868 | 0.76, 1.37 |
| **Antepartum haemorrhage (yes)** |  | **2.03** | **<0.001** | **1.41, 2.91** |
|  |  |  |  |  |
| **Labour and delivery** |  |  |  |  |
| **Place of delivery** |  |  |  |  |
| Health facility |  | 1 |  |  |
| Home |  | 1.36 | 0.204 | 0.84, 2.2 |
| Other |  | **2.12** | **0.012** | **1.18, 3.83** |
| **Rupture of membranes** **≥ 18 hours (yes)** |  | 1.29 | 0.076 | 0.97, 1.71 |
| **Maternal peripartum fever (yes)** |  | 1.07 | 0.690 | 0.74, 1.56 |
| **Mother treated with antibiotics within 24 hrs before/after birth (yes)** |  | **1.45** | **0.001** | **1.15, 1.82** |
| **Mother received antenatal dexamethasone** |  |  |  |  |
| None |  | 1 |  |  |
| 1-3 doses |  | 0.99 | 0.972 | 0.68, 1.44 |
| 4 doses |  | 1.10 | 0.696 | 0.68, 1.76 |
| **Mode of delivery** |  |  |  |  |
| Vaginal unassisted |  | 1 |  |  |
| Vaginal assisted |  | 0.76 | 0.353 | 0.43, 1.35 |
| Caesarean section |  | **0.53** | **<0.001** | **0.42, 0.67** |
|  |  |  |  |  |
| **Neonatal factors** |  |  |  |  |
| **Gender (female)** |  | **1.26** | **0.032** | **1.02, 1.56** |
| **Birth weight (<1.5 kg)** |  | **7.02** | **<0.001** | **5.56, 8.86** |
| **Head circumference on admission (<33 cm)** |  | **2.72** | **<0.001** | **2.14, 3.44** |
| **Length on admission (<45 cm)** |  | **3.43** | **<0.001** | **2.68, 4.39** |
| **Gestational weeks** |  |  |  |  |
| 37-42 |  | 1 |  |  |
| <28 |  | **20.2** | **<0.001** | **12.9, 31.7** |
| 28-<32 |  | **3.7** | **<0.001** | **2.72, 4.94** |
| 32-<37 |  | 1.28 | 0.102 | 0.95, 1.71 |
| 42-45 |  | 1.23 | 0.538 | 0.03, 0.13 |
| **Congenital anomalies (yes)** |  | **1.79** | **0.004** | **1.20, 2.68** |
| **Congenital heart diseases (yes)** |  | 1.29 | 0.436 | 0.68, 2.47 |
| **Received phototherapy (yes)** |  | 0.85 | 0.127 | 0.68, 1.04 |
| **Birth asphyxia (yes)** |  | **1.61** | **<0.001** | **1.27, 2.03** |
| **Suspected sepsis (yes)** |  | **1.24** | **0.043** | **1.01, 1.54** |
| **Respiratory condition (yes)** |  | **2.10** | **<0.001** | **1.70, 2.60** |
| **Abdominal condition (yes)** |  | **2.19** | **0.003** | **1.32, 3.65** |

| **S3 Table. Multivariable logistic regression analysis of selected factors associated with neonatal mortality among vLBW infants (<1.5kg)** | | | | |
| --- | --- | --- | --- | --- |
| **Risk factors** |  | ***Adjusted OR*** | ***P*** | ***95% CI*** |
| **Maternal factors** |  |  |  |  |
| **Maternal age (>=30)** |  | 1.78 | 0.053 | 0.99, 3.18 |
| **Maternal education** |  |  |  |  |
| Did not complete primary education |  | 1 |  |  |
| Completed only primary school |  | 0.77 | 0.605 | 0.29, 2.06 |
| Completed secondary school |  | 1.01 | 0.936 | 0.36, 3.07 |
| Completed tertiary level education |  | 1.19 | 0.844 | 0.6, 3.43 |
| **Maternal occupation** |  |  |  |  |
| Unemployed or housewife |  | 1 |  |  |
| Petty trader/ labourer |  | 0.80 | 0.547 | 0.38, 1.67 |
| Junior schoolteacher/driver |  | 0.74 | 0.537 | 0.28, 1.92 |
| Intermediate public servant/senior schoolteacher |  | 0.42 | 0.179 | 0.12, 1.49 |
| Senior public servant/ professionals/ large scale traders |  | 0.32 | 0.084 | 0.09, 1.16 |
| **Marital status** |  |  |  |  |
| Single |  | 1 |  |  |
| Divorced |  | 1 (omitted) | - | - |
| Married |  | 0.748 | 0.589 | 0.26, 2.20 |
| **Number of stillbirths** |  |  |  |  |
| None |  | 1 |  |  |
| One |  | 2.27 | 0.209 | 0.63, 8.12 |
| Two or more |  | 1.70 | 0.458 | 0.42, 6.94 |
| **Antenatal clinic visits** |  |  |  |  |
| Zero to three |  | 1 |  |  |
| Four to seven |  | 0.94 | 0.859 | 0.52, 1.74 |
| Eight or more |  | 0.34 | 0.158 | 0.05, 1.51 |
| **Number of foetuses** |  |  |  |  |
| 1 |  | 1 |  |  |
| 2 |  | 1.34 | 0.346 | 0.73,2.48 |
| 3- 5 |  | 0.96 | 0.941 | 0.35,2.62 |
| **Pregnancy induced hypertension (yes)** |  | 1.19 | 0.629 | 0.59, 2.38 |
| **Antepartum haemorrhage (yes)** |  | 1.73 | 0.270 | 0.65, 4.57 |
|  |  |  |  |  |
| **Labour and delivery** |  |  |  |  |
| **Place of delivery** |  |  |  |  |
| Health facility |  | 1 |  |  |
| Home |  | 0.93 | 0.918 | 0.26, 3.40 |
| Other |  | 0.99 | 0.990 | 0.12, 7.72 |
| **Prolonged rupture of membranes ≥ 18 hours (yes)** |  | 1.66 | 0.157 | 0.82, 3.33 |
| **Maternal peripartum fever (yes)** |  | 1.27 | 0.606 | 0.51, 3.12 |
| **Mother treated with antibiotics within 24 hrs before/after birth (yes)** |  | **2.83** | **0.002** | **1.46, 5.50** |
| **Mother received antenatal dexamethasone** |  |  |  |  |
| None |  | 1 |  |  |
| 1-3 doses |  | **0.45** | **0.045** | **0.20, 0.98** |
| 4 doses |  | **0.28** | **0.007** | **0.11, 0.70** |
| **Mode of delivery** |  |  |  |  |
| Vaginal unassisted |  | 1 |  |  |
| Vaginal assisted |  | 1.57 | 0.562 | 0.34, 7.27 |
| CS |  | 1.87 | 0.079 | 0.93, 3.79 |
|  |  |  |  |  |
| **Neonatal factors** |  |  |  |  |
| **Gender (female)** |  | **0.53** | **0.023** | **0.31, 0.92** |
| **Head circumference on admission (<33 cm)** |  | 0.49 | 0.632 | 0.03, 8.96 |
| **Gestational weeks (<28 weeks)** |  | **8.59** | **<0.001** | **4.12, 17.93** |
| **Congenital anomalies (yes)** |  | 0.81 | 0.852 | 0.09, 7.31 |
| **Congenital heart diseases (yes)** |  | 0.47 | 0.377 | 0.09, 2.50 |
| **Received phototherapy (yes)** |  | 0.77 | 0.459 | 0.40, 1.51 |
| **Asphyxia (yes)** |  | 1.33 | 0.619 | 0.43, 4.12 |
| **Suspected sepsis (yes)** |  | 1.28 | 0.398 | 0.72, 2.30 |
| **Respiratory condition (yes)** |  | 1.60 | 0.122 | 0.88, 2.89 |
| **Abdominal condition (yes)** |  | 1.84 | 0.274 | 0.62, 5.53 |
| Abbreviation: OR, odds ratio; 95%CI, confidence intervals  Note: Multivariable logistic regression model was used to analyse the factors related to mortality. Variables with low response rate <90% were dropped out in the analysis, including the variables of HIV status, hepatitis B, syphilis, gestational diabetes, and length on admission. Model performance: Log likelihood = -178.40451, R^2^=0.1999, n=326, P<0.001. | | | | |
