# Supplementary material for: Burden of disease and risk factors for mortality amongst hospitalized newborns in Nigeria and Kenya
Source: PLoS One. 2021 Jan 14;16(1):e0244109. doi: 10.1371/journal.pone.0244109 (PMC7808658; doi:10.1371/journal.pone.0244109)
Supplement: Attachment — Submitted filename: Reviewers Comment for manuscript ID.docx [file pone.0244109.s004.docx]

**Reviewers Comment for manuscript ID: PONE-D-20-18123**

**1) Abstract:** It is adequate, well written and explain according to objective.

**2) Introduction:** Very well describe the problem statement but comparative data on other middle income countries are missing. Data from other middle income countries can be describe the severity of neonatal health problem. Para 4 can be reduced.

**3) Methods:**

Study was conducted at different setting (at secondary and tertiary care level) at different countries.

Following question are not clear in methodology

@ Was the admission policy of neonate was same?

@ Who admit the neonates at secondary/ tertiary care level? (Consultant/ Resident)

@ Was the same practice at all NNU?

@ If different persons admitted the neonates, Clinical diagnosis may vary.

@What was the policy for outborn (Home delivered or other facility delivered) neonates?

@Was they admitted with inborn ( same hospital delivered) or at other designated place?

@ What clinical criteria were used to suspect sepsis? You mention only maternal history of premature rupture of membrane and peripartum fever. What about other clinical findings in neonates like lethargy, Sclerema and so on….?

@What about sepsis screen like CRP, micro ESR, IT ratio……?

@What about isolation of Microorganisiam?

@What Respiratory clinical diagnosis were included in respiratory condition? Explain……

@Other than necrotising enterocolitis, what are the other abdominal conditions were included? Explain……..

@How you define birth asphyxia in home delivered neonates?

@What laboratory and radiology investigations were used in diagnosis in study population?

**Ethical approval:** Taken

**Consent of parents:** Taken

**Statistical analysi**s: adequate and proper.

**Results:** Results are properly explain according to objectives.

However, in morbidity and mortality in infants (Table 4)

Jaundice : Serum bilirubin not mention

How many exaggerated physiological, pathological (Rh/ABO incompatibility).

Details about congenital anomalies not mention that leads to death.

How many neonates had life threatening congenital malformations like trachea-esophageal fistula, diaphragmatic hernia or life threatening cardiac malformation like hypoplastic left or right heart syndrome that leads to death in early neonatal period?

Was there any follow up after discharge to say as morbidity?

**Discussion:**

Very well explain and appropriate.

**Conclusion:** Appropriate and concise.

**Funding:** Mention

**Competing interest:** mention

**References:** adequate and recent

**Tables and figure:** Wel explained
