# Supplementary material for: Burden of disease and risk factors for mortality amongst hospitalized newborns in Nigeria and Kenya
Source: PLoS One. 2021 Jan 14;16(1):e0244109. doi: 10.1371/journal.pone.0244109 (PMC7808658; doi:10.1371/journal.pone.0244109)
Supplement: Attachment — Submitted filename: Comments on the Manuscript.docx [file pone.0244109.s005.docx]

**Comments on the Manuscript: PONE-D-20-18123**

**Introduction**

1. The authors need to provide a detail explanation of the scope of the research
2. The authors need to outline the expected advantages of the research.

**Methods**

3. Study setting: Why were only these facilities chosen? Could other health facilities in different parts of the countries been included?
4. Are these facilities representative of all neonatal units in Nigeria and Kenya?
5. What was the inclusion and exclusion criteria for the study?
6. Why were newborns aged above 48 hours excluded from the study?
7. Missing data: The authors need to explain the average percentage of missing data for each variable and justify that removing such data does not affect the analysis.

**Results**

8. Table 3: The authors indicated that 2182 neonates had birth weight recorded. However, percentages are estimated based on 2181 (Section: Newborn admission characteristics). Kindly check and correct the inconsistency.
9. Page 10: 77.9% (81/104) mortality among extremely LBW newborns. However, this number is included in the mortality rate of VLBW newborns (222/472). The authors need to separate these two categories of birthweight to provide a better appreciation of the mortality risk of newborns between 1kg and 1.5kg.
10. Figure 3: Why is the number of categories for some variables (example birthweight) different from table 3? Explain as part of the data analysis why the categories for the regression models different from the descriptive analysis.
