# Supplementary material for: Burden of disease and risk factors for mortality amongst hospitalized newborns in Nigeria and Kenya
Source: PLoS One. 2021 Jan 14;16(1):e0244109. doi: 10.1371/journal.pone.0244109 (PMC7808658; doi:10.1371/journal.pone.0244109)
Supplement: Attachment — Submitted filename: Reviewers Comment for manuscript ID R1.docx [file pone.0244109.s007.docx]

**Reviewers Comment for manuscript ID: PONE-D-20-18123R1**

**Abstract:** It is adequate, well written and explain according to objective.

**Introduction:** Correction done according to comments but still I feel, though this study focus to generate the evidences from sub-Saharahan Africa, comparative data of other middle income countries will is important as a readers view.

**Methods:** Adequate and clear the idea.

**Ethical approval:** Taken

**Consent of parents:** Taken

**Statistical analysi**s: adequate and proper.

**Results:** Results are properly explained according to objectives.

**Discussion:**

Very well explain and appropriate.

**Conclusion:** Appropriate and concise.

**Funding:** Mention

**Competing interest:** mention

**References:** adequate and recent

**Tables and figure:** Wel explained
